# Optimizing Hydrogen Production: Influence of Promoters in Methane Decomposition on Titania-Modified-Zirconia Supported Fe Catalyst

**DOI:** 10.1021/acsomega.4c00729

**Published:** 2024-04-25

**Authors:** Ahmed S. Al-Fatesh, Dharmesh M. Vadodariya, Mohammed O. Bayazed, Ahmed I. Osman, Ahmed Aidid Ibrahim, Anis Hamza Fakeeha, Yousef M. Alanazi, Ahmed E. Abasaeed, Rawesh Kumar

**Affiliations:** † Chemical Engineering Department, College of Engineering, King Saud University, P.O. Box 800, Riyadh 11421, Saudi Arabia; ‡ Department of Chemistry, Indus University, Ahmedabad, Gujarat 382115, India; § School of Chemistry and Chemical Engineering, Queen’s University Belfast, Belfast BT9 5AG, Northern Ireland, U.K.

## Abstract

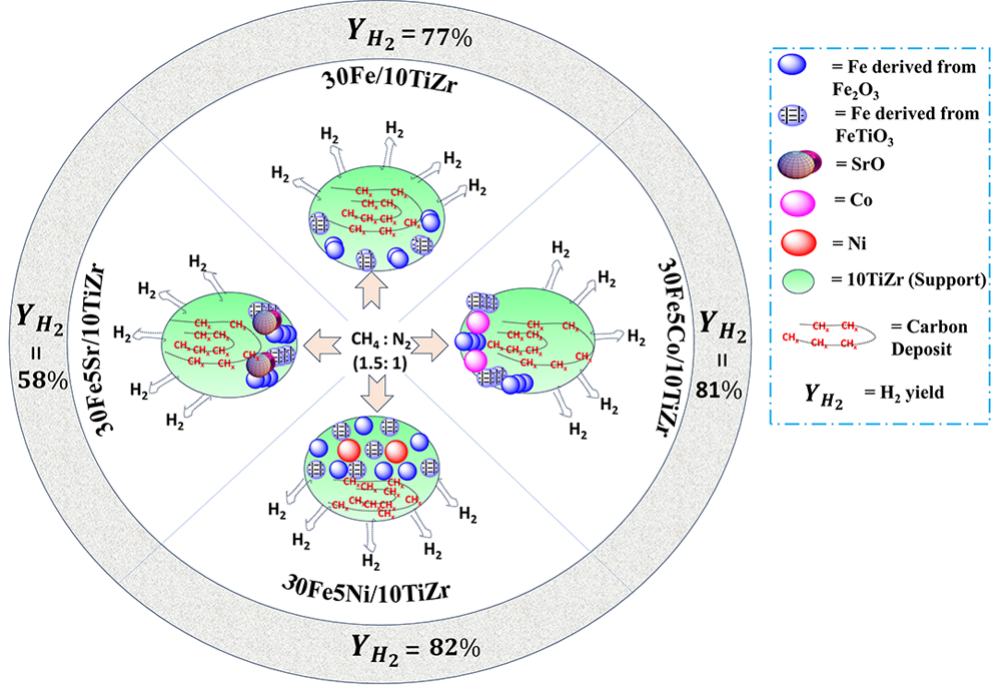

This study addresses the pivotal challenge of hydrogen production through methane decomposition, offering a pathway to achieving clean energy goals. Investigating the utilization of titania-modified zirconia dual redox supports (10TiZr) in iron or doped iron-based catalysts for the CH_4_ decomposition reaction, our research involves a thorough characterization process. This includes analyses of the surface area porosity, X-ray diffraction, Raman-infrared spectroscopy, and temperature-programmed reduction/oxidation. The observed sustained enhancement in catalytic activity over extended durations suggests the *in situ* formation of catalytically active sites. The introduction of Co or Ni into the 30Fe/10TiZr catalyst leads to the generation of a higher density of reducible species. Furthermore, the Ni-promoted 30Fe/10TiZr catalyst exhibits a lower crystallinity, indicating superior dispersion. Notably, the cobalt-promoted 30Fe/10TiZr catalyst achieves over 80% CH_4_ conversion and H_2_ yield within 3 h. Additionally, the Ni-promoted 30Fe/10TiZr catalyst attains a remarkable 87% CH_4_ conversion and 82% H_2_ yield after 3 h of the continuous process.

## 1 Introduction

Methane, being 28 times more potent than CO_2_ as a greenhouse gas, poses a significant environmental challenge, necessitating effective catalytic solutions.^1^ The catalytic decomposition of CH_4_ into carbon and H_2_ not only addresses this challenge but also presents environmentally favorable attributes. Notably, this process avoids the production of hazardous greenhouse gases, exhibits moderate endothermic characteristics, and yields solid carbon as a byproduct. The strategic separation of hydrogen gas from carbon has emerged as a practical approach, contributing to the quest for pure hydrogen.

The pursuit of pure hydrogen energy through CH_4_ depletion stands as a paramount scientific endeavor, demanding impactful research efforts. The appropriate catalyst, reaction conditions, and reactor types play a crucial role in achieving pure hydrogen with high yield through CH_4_ decomposition (CH_4_(*g*) → C(s) + 2H_2_(*g*)).^2,3^ While activated carbon, particularly enriched with defects or surface oxides, has shown promise in CH_4_ decomposition,^2,3^ metal-based catalysts, including Co, Fe, and Ni, have garnered significant attention in large-scale catalyst development within the industry.^4−6^ Furthermore, strontium hexaferrite, when subjected to a CH_4_ stream, undergoes reduction to Fe_2_O_3_ → Fe → Fe_3_C,^7^ with Fe_3_C acknowledged as a catalyst for CH_4_ decomposition reactions.^8^

Metals like cobalt, crucial for exposing a maximum density of active sites to reactants, benefit from dispersion over thermally sustainable supports, such as alumina, silica, zirconia, and titania. Co–Al mixed oxides, derived from hydrotalcite with a Co/Al mol ratio of 2–3, demonstrated significant promise by enriching the easily reducible Co_2_AlO_4_ phase. This led to a notable achievement of high initial methane conversion (>80% at 750 °C).^9^ However, when the Co/Al ratio exceeded 3, severe sintering and catalyst deactivation were observed. The addition of Sr over Co/Al_2_O_3_, forming Sr_4_Al_14_O_25_, effectively restrained the generation of CoAl_2_O_4_ species, resulting in higher activity and stability for the methane decomposition reaction.^10^ The catalyst showed 80% CH_4_ conversion, 90% H_2_ selectivity, and 10% CO_2_ selectivity at 750 °C and 18000 mL h^–1^ g^–1^ GHSV.

Iron dispersed over silica, silica–alumina, and alumina was found to be completely inactive, active, and highly active, respectively.^11,12^ An iron-alumina catalyst prepared using the wet agglomeration method showed 80% conversion continuously up to 160 h under a fluidized test.^11^ Again, the strontium-promoted Fe-based catalyst supported by only silica was inactive. When support was changed to silica–alumina (Si/Al = 20/80), the catalyst became highly active (average H_2_ yield of 80% over a 330 min period of reaction), and coke formation was markedly suppressed (up to 26%).^12^ Among the dual active sites, Ni dispersed over iron aluminate and Ni dispersed over ferrate come up with a Ni–Fe alloy having strong metal–support interaction.^13,14^ FeAl_2_O_4_-supported nickel catalyst exhibited 21% CH_4_ conversion at 575 °C during the 300 min time on stream.^13^ Nickel ferrite showed about 48.5% CH_4_ conversion with 97.70 × 10^–5^ mol H_2_ g^–1^ min^–1^ hydrogen formation rate.^14^ Sun et al. prepared a zeolite (high-silica)-supported Ni catalyst, which was further doped with Fe and Zn. The Ni–Fe alloy phase stabilized the catalytic active sites, whereas Zn induced markable carbon diffusion to avoid excessive deposition. The catalyst showed ∼60% CH_4_ conversion up to 6 h at 670 °C.^15^

High-temperature catalytic application is unsuitable for redox supports such as TiO_2_ and ZrO_2_ due to the phase transition of anatase-rutile TiO_2_ phases and monoclinic-tetrahedral ZrO_2_ phases. Fe supported on TiO_2_ exhibited poor activity and stability and was found to be unsuitable for the CH_4_ decomposition reaction.^16^ However, using NiO in a double mole ratio with TiO_2_ (Ni/Ti = 2) resulted in improved catalyst performance. The catalyst showed a constant 65% H_2_ volume up to 500 min at 700 °C.^17^ It should be noted, however, that using a concentration of active sites higher than the support may not be recommended for catalytic application. A ZrO_2_-supported Ni catalyst showed a 20% H_2_ yield after 230 min.^18^ However, when ZrO_2_ was modified by lanthana, the catalytic activity increased to (25% H_2_ yield after 230 min). Ni stabilized over tungsten-zirconia support has strong metal–support interaction and achieved more than 90% H_2_ yield up to 230 min.

Upon an extensive literature review, it was found that dual redox metal oxide supports have not been thoroughly investigated for iron- or doped iron-based catalysts in CH_4_ decomposition reactions. Reports suggest that the synergy between Fe and Zr can enhance the dispersion of Fe_2_O_3_.^19^ Thus, a support predominantly composed of Zr may provide improved dispersion of Fe_2_O_3_. In this study, 10 wt % titania-modified zirconia (10TiZr) support and 30 wt % Fe (30Fe/10TiZr) were prepared. The catalyst samples were characterized through surface area porosity, X-ray diffraction, Raman-infrared spectroscopy, and temperature-programmed reduction/oxidation techniques. The fine-tuning between characterization results and catalytic activity put significant insight into the use of titania–zirconia for supporting the active sites and stabilizing promoters toward the CH_4_ decomposition reaction. The role of different promoters on the surging and dispersion of active sites (under hydrogen before the reaction and under CH_4_ during the reaction) over the catalyst and their subsequent catalytic performance are found to be closely related. The valuable outcome of current research will significantly advance the iron-based catalysis toward the CH_4_ decomposition reaction.

## 2 Experimental Section

### 2.1 Materials

Fe (NO_3_)_3_·9H_2_O [Purity 99%, Loba Chemie], Ni (NO_3_)_2_·6H_2_O (Purity 98%, Alfa Aesar), Co (NO_3_)_2_ (Purity 99%, Aldrich), Sr (NO_3_)_2_·9H_2_O (Purity 99%, Sigma-Aldrich, Steinheim, Germany), titanium dioxide (AEROXIDE TiO_2_ P25, Evonik Industries AG, Germany), Zr (OH)_2_ ((Daiichi Kigenso (Osaka, Japan)), and 10 wt % TiO_2_–90 wt % ZrO_2_ (Daiichi Kigenso Kagaku Kogyo Co) were used.

### 2.2 Catalysts Preparation

The 10 wt % titania and 90 wt % zirconia are mixed mechanically in a mortar pestle and then calcined at 600 °C for 3 h. The titania-modified zirconia support is abbreviated as 10TiZr. The 30 wt % equivalent solution of iron nitrate, 5 wt % equivalent solution of promoters’ nitrates (nickel nitrate, cobalt nitrate, and strontium nitrate), and 10TiZr support are mixed under magnetic stirring at 80 °C for 2 h to ensure comprehensive mixing and even dispersion of the precursor on the support. The resulting mixture is dried overnight at 120 °C and calcined at 600 °C for 3 h. Finally, the catalyst material is crushed into a powder for chemical reactions.

### 2.3 Catalyst Performance Evaluation

The methane decomposition reaction is carried out by 1.5 CH_4_:1 N_2_ feed gas (flow rate of 20 mL/min, gas hourly space velocity of 8000 mL/(h·g_cat_)) over 0.15 g of catalyst packed in a fixed-bed stainless steel tubular reactor (9.1 mm inner diameter and a length of 30 cm). The temperature of the catalyst bed is monitored by a K-type thermocouple, which is positioned at the center of the catalyst bed. Before the CH_4_ decomposition reaction, the catalyst is reduced under H_2_ (flow rate of 40 mL/min) at 700 °C for 60 min. After reducing the catalyst, the reactor’s temperature is adjusted to 800 °C under a nitrogen flow (15 mL/min) for a methane decomposition reaction. The reactor outlet composition is analyzed using online gas chromatography with a thermal conductivity detector (TCD). CH_4_ conversion and hydrogen yield are calculated using the following formulas:
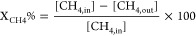
1
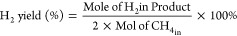
2where [CH_4,in_] = initial methane content in the feed and [CH_4,out_] = methane content that remains in the product.

### 2.4 Catalyst Characterization

The textural property of the fresh catalyst samples is analyzed by N_2_ physisorption using a Micromeritics TriStar II 3020 instrument. Before analysis, the catalyst sample is degassed at 250 °C for 3 h. The Brunauer–Emmett–Teller (BET) and Barret–Joyner–Halenda (BJH) methods were used to estimate the specific surface area and pore volume-pore size, respectively. X-ray diffraction (XRD) patterns were obtained using a Miniflex Rigaku diffractometer using CuKα radiation operating at 40 kV voltage and 40 mA current. The phase analysis is carried out by X’pert high, scored, and equipped with the JCPDS database. Temperature-programmed reduction/oxidation (TPR/TPO) was conducted on fresh and spent catalysts using Micromeritics AutoChem II 2920 equipped with a thermal conductivity detector (TCD). TPR involved treating 70 mg of the catalyst with argon at 150 °C, followed by a temperature ramp to 1000 °C under a H_2_/Ar mixture. TPO measured carbon deposits on spent catalysts in the presence of oxygen, analyzing a temperature range of 50–900 °C. Thermogravimetric (TGA) analysis of the 10–16 mg spent catalyst samples was carried out by Shimadzu TGA-51 (Shimadzu Corporation, Kyoto, Japan) in the temperature range from room temperature to 1000 °C (at 20 °C/min temperature ramp) under O_2_. The mass change in the spent catalyst sample was constantly monitored during heat treatment. Raman spectroscopy techniques are employed over fresh and spent catalysts using a Laser Raman (NMR-4500) spectrometer (JASCO, JAPAN) under a beam excitation wavelength of 532 nm.

## 3 Result and Discussion

### 3.1 Characterization Results

**Figure 1:**
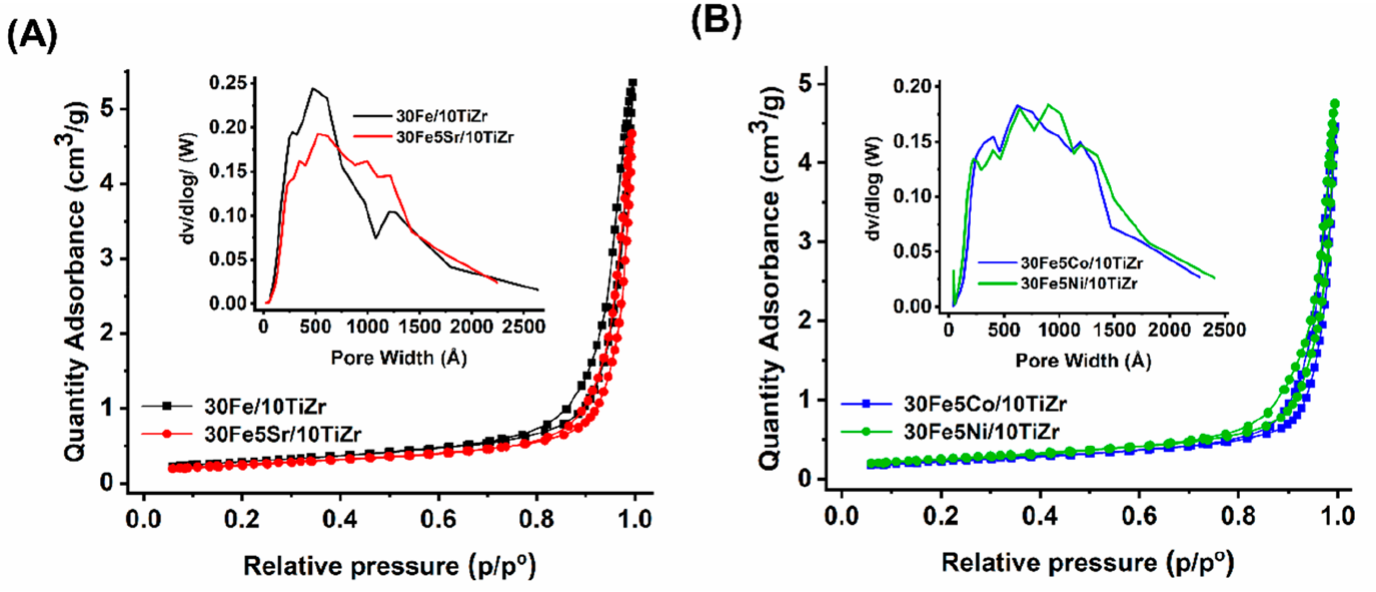
N_2_-adsorption isotherms and porosity distributions of (A) 30Fe/10TiZr and 30Fe5Sr/10TiZr and (B) 30Fe5Co/10TiZr and 30Fe5Ni/10TiZr.

The N_2_-adsorption isotherm and porosity distribution of 30Fe/10TiZr and 30Fe5M/10TiZr (M = Sr, Co, and Ni) catalysts are shown in Figure 1A,B. The surface area, pore volume, and pore diameter of each catalyst are shown in Table 1. All catalyst samples show a type IV isotherm with an H1 hysteresis loop having sharp inflection beyond 0.8 relative pressure. It indicates the presence of the capillary architects of mesopores.^20^ 10TiZr supported iron catalyst has a 23 m^2^/g surface area, 0.19 cm^3^/g pore volume, and 31.2 nm pore diameter. Upon the addition of 5 wt % promotors (Sr, Co, and Ni) over 30Fe/10TiZr catalyst, the surface area and pore volume decreased. It indicates the deposition of promoter oxide crystallite in the mesopores. d*V*/dlog(*w*) versus pore diameter plot shows a bimodal pore size distribution over 30Fe/10TiZr catalyst and multimodal pore size distribution over promoted 30Fe/10TiZr catalysts.

**Table 1:** Surface Area, Pore Volume, and Pore Diameter of Each Catalyst

Catalyst	Surface Area (m^2^/g)	Pore volume (cm^3^/g)	Pore Diameter (nm)
30Fe/10TiZr	23	0.19	31.2
30Fe5Sr/10TiZr	20	0.16	35.2
30Fe5Co/10TiZr	18	0.16	35.8
30Fe5Ni/10TiZr	20	0.17	32.1

**Figure 2:**
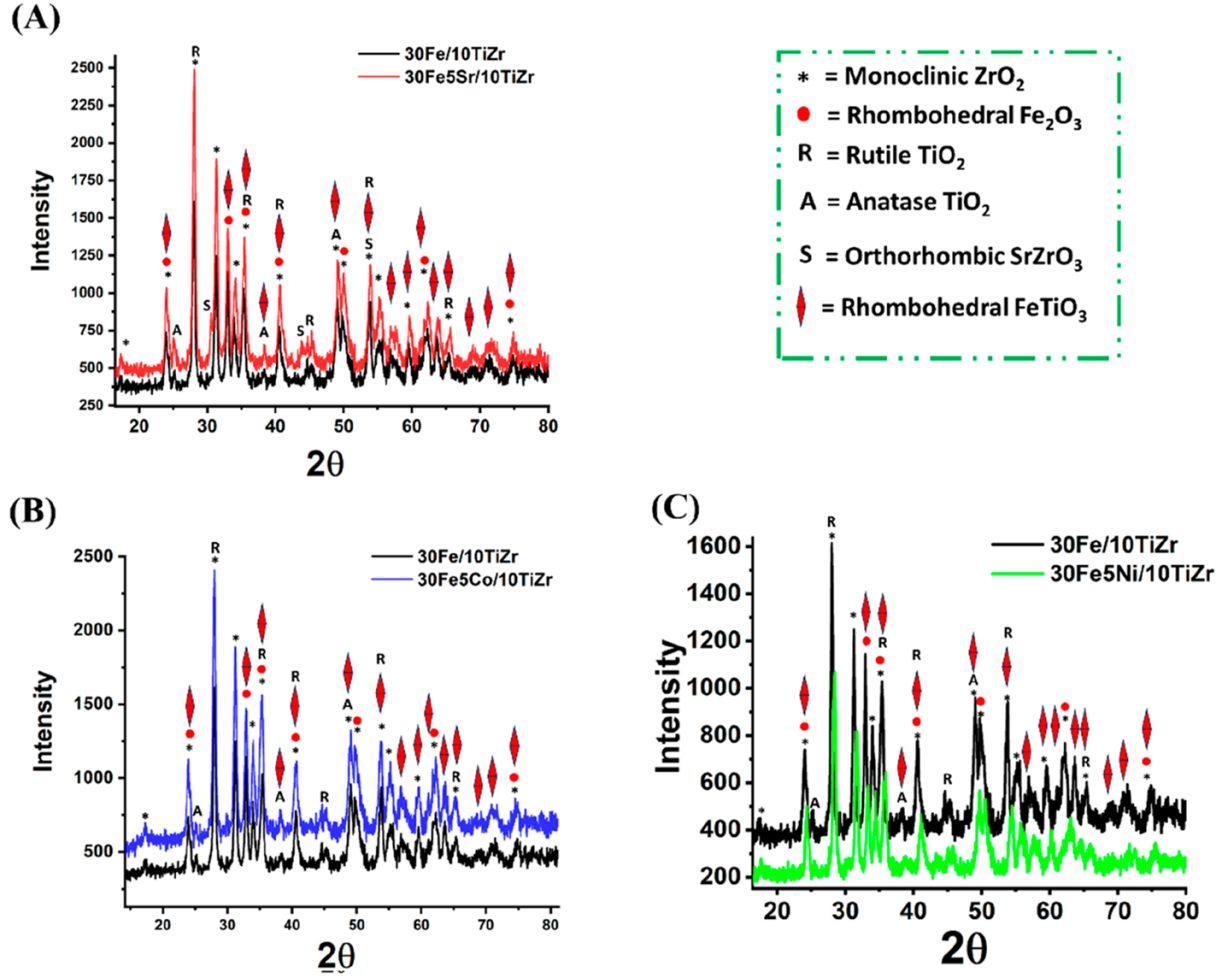
X-ray diffraction patterns of (A) 30Fe/10TiZr and 30Fe5Sr/10TiZr, (B) 30Fe/10TiZr and 30Fe5Co/10TiZr, and (C) 30Fe/10TiZr and 30Fe5Ni/10TiZr.

X-ray diffraction studies of 30Fe/10TiZr and 30Fe5M/10TiZr (M = Sr, Co, Ni) catalysts are shown in Figure 2. The 30 wt % iron supported on titania-modified zirconia is highly crystalline. It has a monoclinic zirconia phase (at Bragg’s angle 2θ = 17.2°, 23.96°, 27.95°, 31.25°, 33.93°, 35.41°, 38.3°, 40.59°, 49.05°, 53.8°, 55.24°, 56.83°, 59.57°, 62.37°, 65.38°, 75.01°; JCPDS reference number: 00-001-0750), anatase TiO_2_ phase (at Bragg’s angle 2θ = 25.28°, 38°, 48.7°; JCPDS reference number: 00-001-0562), rutile TiO_2_ phase (at Bragg’s angle 2θ = 27.95°, 35.42°, 41.24°, 54.3°; JCPDS reference number 01-076-0649), and the rhombohedral Fe_2_O_3_ phase (at Bragg’s angle 2θ = 23.96°, 32.93°,35.42°, 40.59°, 49.79°, 62.37°, 75.01°; JCPDS reference number 01-084-0307). The mixed oxide phase of iron and titanium oxide, named the rhombohedral FeTiO_3_ phase, is also formed. The diffraction pattern of the rhombohedral FeTiO_3_ phase is generally merged with the rhombohedral Fe_2_O_3_ and TiO_2_ phases at Bragg’s angle 2θ = 23.96°, 32.93°, 35.42°, 38.30°, 40.59°, 49.05°, 53.80°, 56.84°, 59.57°, 62.37°, 63.60°, 65.39°, 75.01° (JCPDS reference number 01-075-1212). The crystalline peak intensity of all the discussed phases is increased upon adding 5 wt % Co or Sr over 30Fe/10TiZr catalyst. It indicates that cobalt or strontium addition induces higher crystallinity in the existing phases. 30Fe5Sr/10TiZr catalyst nurtures the orthorhombic strontium zirconium oxide phase (at Bragg’s angle 2θ = 30.57, 43.88, 54.3; JCPDS reference number 01-070-0695) additionally. The diffraction pattern due to the 5 wt % addition of Ni over 30Fe/10TiZr is noticeable. The intensity of diffraction peak patterns decreases and shifts to a higher Bragg’s angle over the 30Fe5Ni/10TiZr catalyst. The unpromoted catalyst compresses the unit cell of the 30Fe5Ni/10TiZr catalyst.

**Figure 3:**
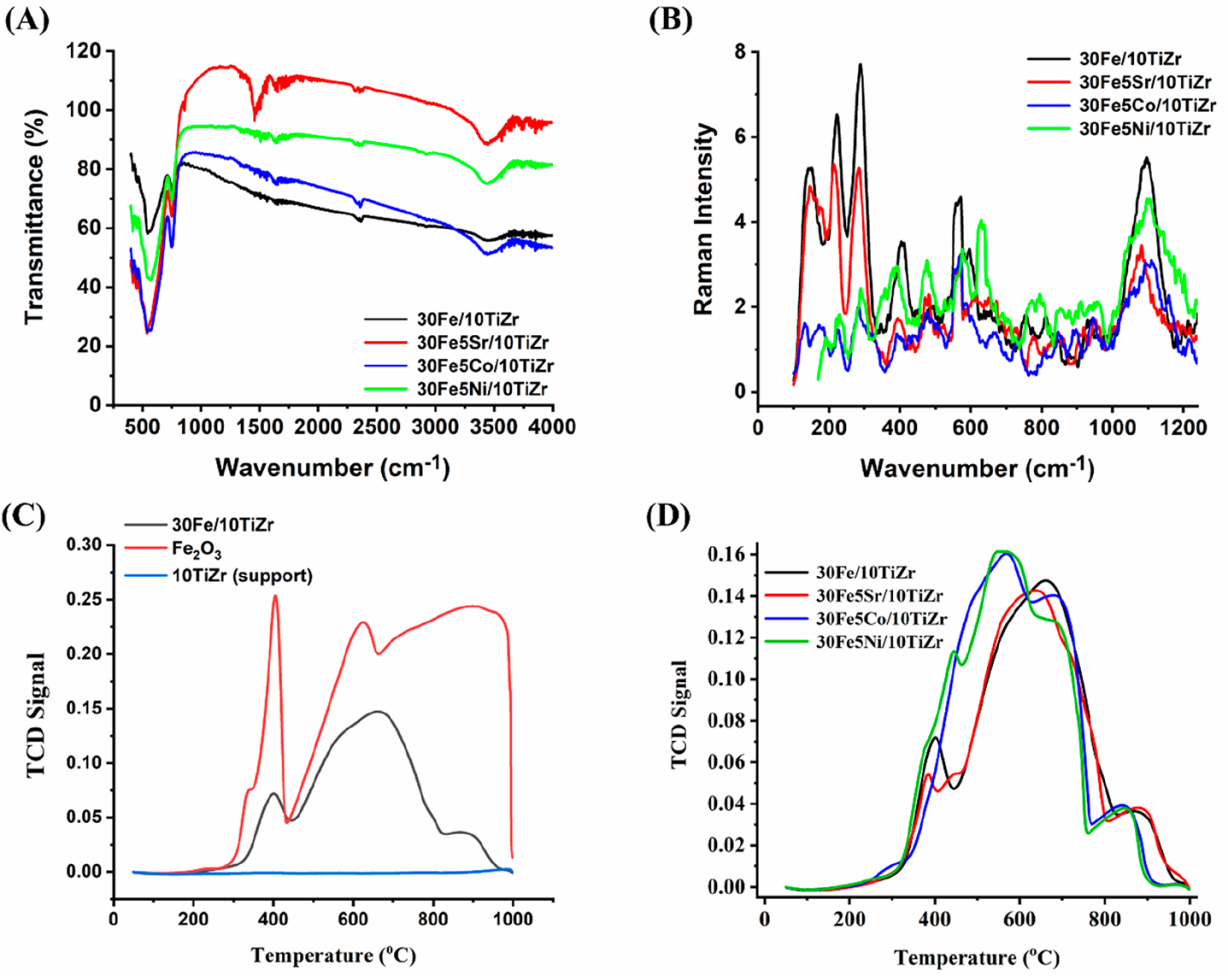
(A) Fourier transform infrared spectra of 30Fe/10TiZr and 30FeM/10TiZr (M = Sr, Co, Ni) catalysts. (B) Raman spectroscopy of 30Fe/10TiZr and 30FeM/10TiZr (M = Sr, Co, Ni) catalysts. (C) H_2_-temperature-programmed reduction profile of Fe_2_O_3_, 10TiZr, and 30Fe/10TiZr. (D) H_2_-temperature-programmed reduction profile of 30Fe/10TiZr and 30FeM/10TiZr (M = Sr, Co, Ni) catalysts.

Figure 3A exhibits the Fourier transform infrared spectra of the 30Fe/10TiZr and 30Fe5M/10TiZr (M = Sr, Co, Ni) catalysts. Iron supported over titania-modified zirconia catalyst and metal (M = Sr, Co, Ni) promoted iron supported over titania-modified zirconia catalyst has a characteristic stretching vibration band for Zr–O at 748 cm^–1^.^21,22^ The vibration spectra of the 30Fe5Sr/10TiZr catalyst have additional peaks of about 859 and 1464 cm^–1^, which are attributed to ionic CO_3_^2–^ species.^23,24^ The formation of SrCO_3_ by the interaction of SrO and CO_2_ (from humid air) is very common.^25^

The Raman spectra of fresh 30Fe/10TiZr and 30Fe5M/10TiZr (M = Sr, Co, and Ni) are shown in Figure 3B. Monoclinic ZrO_2_ is Raman active and produces an oxygen vacancy. But interestingly, the Raman bands for monoclinic ZrO_2_ are either absent or diffused. In nanocrystalline ZrO_2_, most oxygen vacancies act as media for magnetic interaction between Zr ions.^26^ The single charge oxygen vacancy reduces Zr^4+^ to Zr^3+^, creating a Zr vacancy. Thermodynamically, the Zr vacancy is less favorable than the oxygen vacancy. As observed from XRD, the catalyst surface contains iron-related phases having Fe^2+^ and Fe^3+^ oxidation states. In the current catalyst system, the oxygen vacancy created by Fe^3+^ and Fe^2+^ is much more dominant than the Zr vacancy. So, Raman spectra for the monoclinic zirconia phase are not observed prominently over the current catalyst system. 30Fe/10TiZr catalyst shows Raman bands of about 223, 291, and 410 cm^–1^ for relevant vacancies due to the coexistence of Fe^2+^ and Fe^3+^ oxidation states over the catalyst.^27,28^ The Raman bands at about 148 cm^–1^, 418 cm^–1^, and 600 cm^–1^ are also present over 30Fe/10TiZr. In the literature, the Raman bands at 144 ± 8 cm^–1^, 600 ± 7 cm^–1^, and 418 ± 10 cm^–1^ are also reported for nanophase TiO_2_, and these peaks are highly dependent on annealing temperature.^29^ The band at 1320 cm^–1^ is due to two-magnon scattering arising from the interaction of two magnons created on close antiparallel spin sites. It is interesting to note that these peaks are suppressed noticeably upon the addition of promoters like Sr and Co. The Raman peak intensity is suppressed extensively in the case of 30Fe5Co/10TiZr. It seems that vacancies over the 30Fe/10TiZr catalyst are the preferred sites for promoter interaction, and after the addition of promoters, these sites are preferentially occupied.

The reduction profile of catalysts is studied by H_2_-temperature-programmed reduction (H_2_-TPR) and shown in Figure 3C,D. It is reported that titanium dioxide is partially reduced to TiO_2–*x*_ under hydrogen at high temperatures (above 500 °C).^30^ However, the titania-modified zirconia support has no peak in H_2_-TPR, indicating the nonreducibility of the support. The bulk Fe_2_O_3_ material gives three reductive peaks at 405 °C, 621 °C, and ∼900 °C. These peaks are attributed to the sequential reduction of Fe_2_O_3_ → Fe_3_O_4_ → FeO → Fe.^31^ Iron dispersed over a titania–zirconia support also had reduction peaks in the same temperature range. That means the same type of reducible pattern is presented in the current catalyst system as bulk Fe_2_O_3_ material.

Interestingly, the last reduction peak in bulk Fe_2_O_3_ material is broad, but in 30Fe/10TiZr, the last peak has diffuse intensity. That means the FeO, which interacts with the support (in the 30Fe/10TiZr catalyst), is less reducible than the free FeO (in bulk Fe_2_O_3_). Only a small portion of FeO is converted to Fe under the H_2_ stream. The reducibility pattern of strontium-promoted 30Fe/10TiZr is more or less the same as that of the 30Fe/10TiZr catalyst. The 30Fe5Co/10TiZr catalyst had an additional reduction peak at about 560 °C temperature. It is reported that the bulk Co_3_O_4_ material has a single symmetric reduction peak of about 327 °C (for reduction of Co_3_O_4_ to Co) under H_2_.^32^ In XRD, we have not observed the phases related to Co. It may be possible that highly dispersed cobalt oxide (CoO_*x*_) remains unreachable for XRD detection. The reduction peak at a high temperature of about 560 °C is attributed to the reduction of small CoO_*x*_ clusters with relatively stronger interaction.^32^ 30Fe5Ni/10TiZr catalyst has additional reducible NiO species over the surface. The H_2_-TPR profile of 30Fe5Ni/10TiZr has additional peaks at about 450 and 560 °C. The peak of about 450 °C is attributed to NiO species, which interacts with support with weak attraction. In contrast, the reduction peak at about 560 °C is attributed to NiO species, which interact moderately with the support. The presence of NiO species, which interact strongly with the support, cannot be neglected. The reduction peak of about 830 °C may be attributed to the reduction of “strongly interacting NiO” and “reduction of FeO”.^31^

### 3.2 Catalytic Activity Results

**Figure 4:**
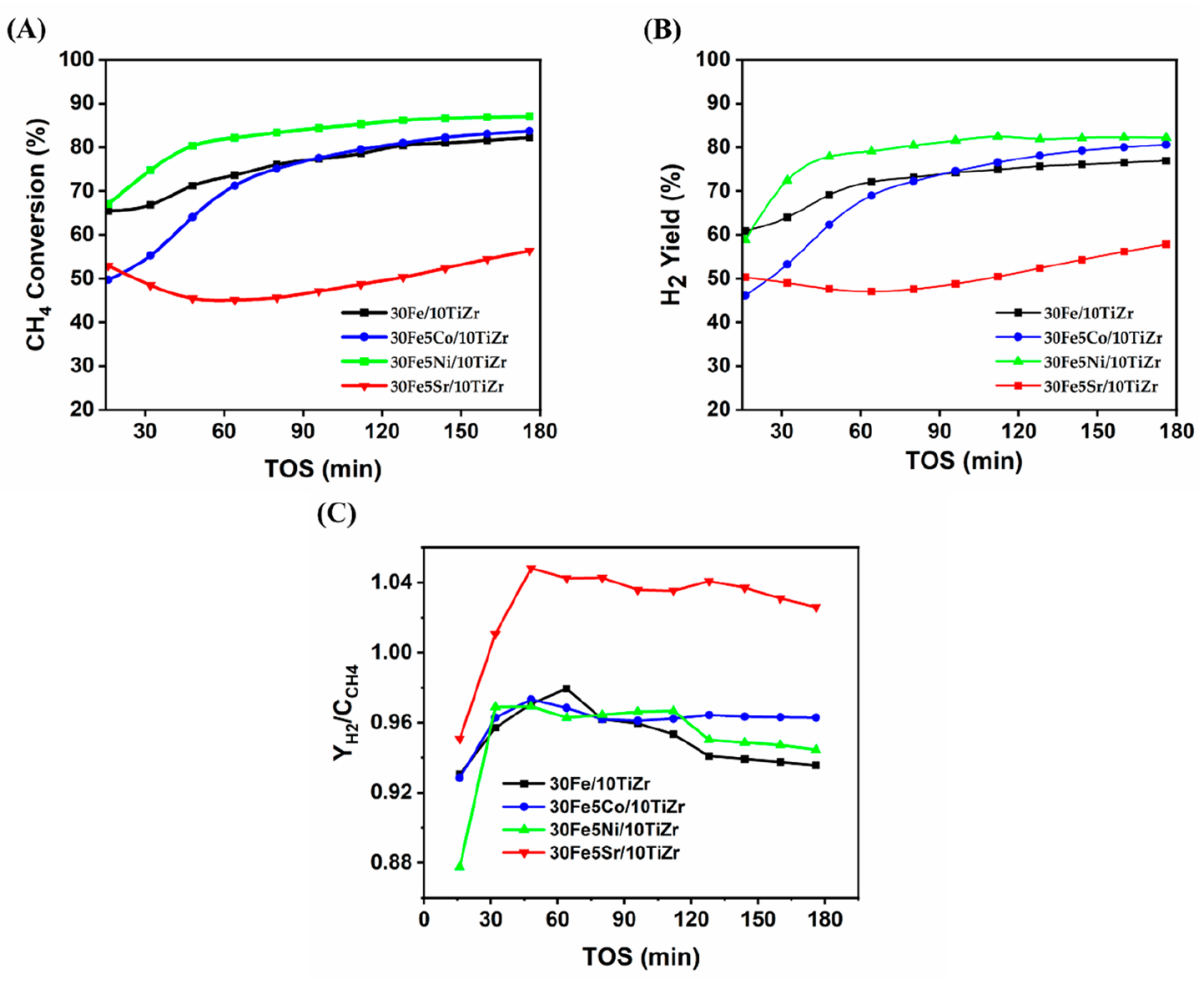
Catalytic activity results of (A) CH_4_ conversion, TOS, (B) H_2_ yield, and (C) *Y*_*H*_2__/*C*_*CH*_4__ vs TOS.

Figure 4 illustrates the catalytic activity of 30Fe/10TiZr and 30Fe5M/10TiZr (M= Sr, Co, Ni) based on CH_4_ conversion, H_2_ yield, and the ratio of hydrogen yield to CH_4_ conversion, (*Y*_*H*_2__/*C*_*CH*_4__). In the current study, the *Y*_*H*_2__/*C*_*CH*_4__ value is observed between 0.93 and 1.02 over 30Fe/10TiZr and 30Fe5M/10TiZr (M = Sr, Co, Ni) catalysts. *Y*_*H*_2__/*C*_*CH*_4__< 2 is explained by the formation of CH_*x*_ over the catalyst surface, whereas high H_2_ yield compared to CH_4_ conversion indicates the accumulation of CH_4_ over the catalyst surface.^33^ All catalysts show an increase in CH_4_ conversion and H_2_ yield after 3 h compared to initial activity. The 30Fe/10TiZr catalyst shows initial 65% CH_4_ conversion, 61% H_2_ yield, and 0.93 *Y*_*H*_2__/*C*_*CH*_4__. After 3 h, the catalyst attains 82% CH_4_ conversion and 77% H_2_ yield without affecting the *Y*_*H*_2__/*C*_*CH*_4__ ratio. After the addition of Co to 30Fe/10TiZr, the catalytic activity initially drops to 50% CH_4_ conversion and 46% H_2_ yield. However, after 3 h of reaction time, the activity significantly increases to 84% CH_4_ conversion and 81% H_2_ yield, exceeding the performance of the unpromoted catalyst. When Sr is added over 30Fe/10TiZr, it results in lower catalytic activity with less than 60% CH_4_ conversion and a H_2_ yield. But it achieves the highest *Y*_H_2__/*C*_CH_4__ ratio during a 3 h time on stream. The catalytic activity of Ni-promoted 30Fe/10TiZr always remains highest compared to others, and it shows an initial 67% CH_4_ conversion, 59% H_2_ yield, and 0.88 *Y*_*H*_2__/*C*_*CH*_4__. The activity progressed continuously, and at the end of 3 h, 87% CH_4_ conversion, 82% H_2_ yield, and 0.94 *Y*_*H*_2__/*C*_*CH*_4__ were found during 3 h of reaction.

### 3.3 Postcharacterization Results

**Figure 5:**
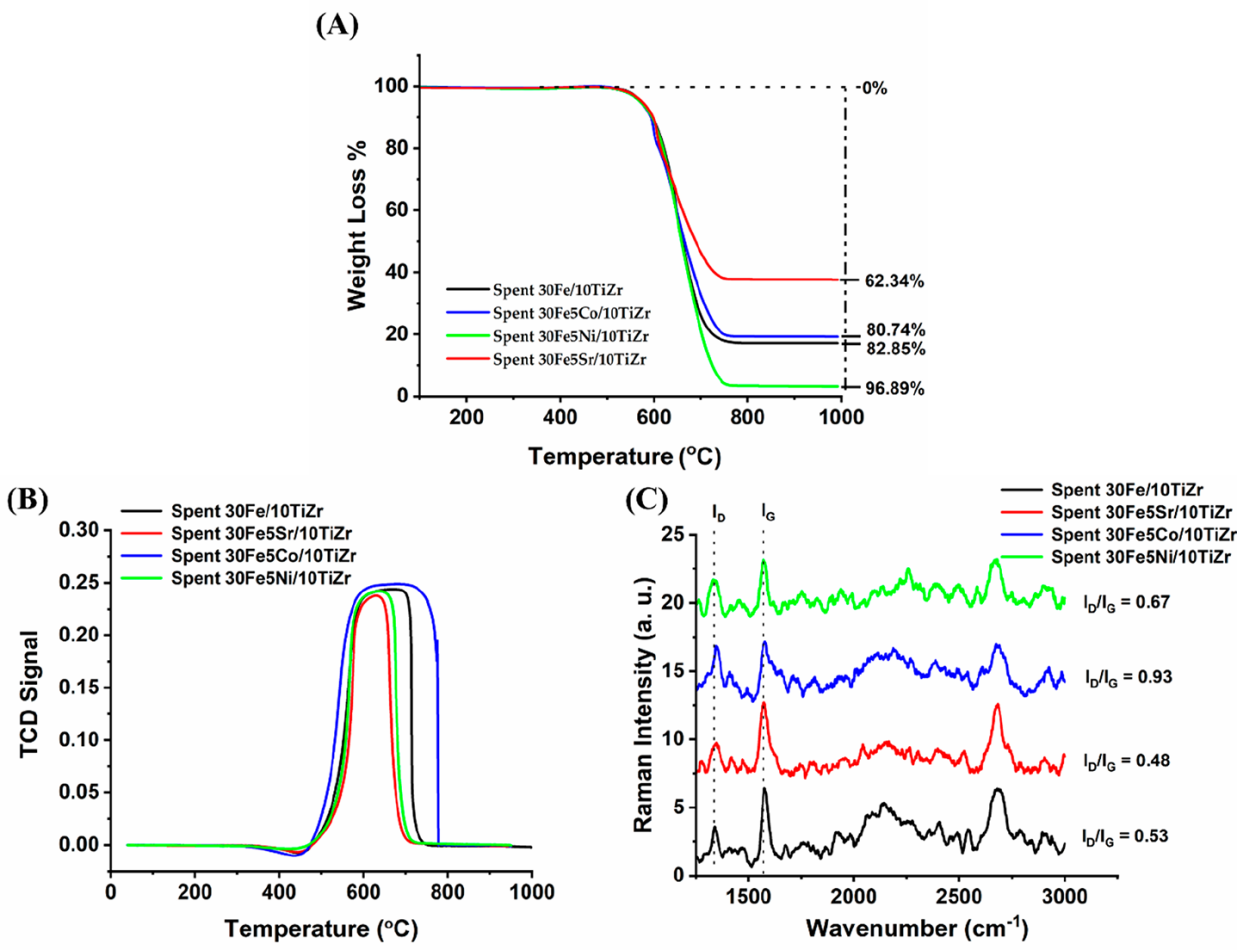
Characterization result of spent 30Fe/10TiZr and spent 30Fe5M/10TiZr (M = Sr, Co, Ni) catalyst: (A) thermogravimetry analysis, (B) O_2_-TPO profile, and (C) Raman spectroscopy.

Figure 4 shows the (A) thermogravimetry analysis, (B) O_2_-TPO profile, and (C) Raman spectroscopy of spent 30Fe/10TiZr and spent 30Fe5M/10TiZr (M = Sr, Co, Ni) catalysts. The thermogravimetry result of spent 30Fe/10TiZr and spent 30Fe5M/10TiZr (M = Sr, Co, and Ni) shows the highest weight loss over Ni promoted 30Fe/10TiZr catalyst and the minimum weight loss over strontium promoted 30Fe/10TiZr catalyst. The weight loss profiles over an unpromoted catalyst (30Fe/10TiZr) and cobalt-promoted catalyst (30Fe5Co/10TiZr) are very close to each other. The net weight loss result in TGA is a combined contribution of weight loss due to the oxidation of carbon deposit and weight gain due to oxidation of reduced species (Fe, Ni, Co). The current catalyst systems have plenty of active sites in a metallic state, which can oxidize under O_2_ below 1000 °C. To study the carbon deposit/carbon type, O_2_-TPO and Raman spectroscopy are more promising than TGA. The O_2_-TPO profile of spent 30Fe/10TiZr and spent 30Fe5M/10TiZr (M = Sr, Co, Ni) is shown in Figure 5B. In O_2_-TPO, the peaks below 500 °C, between 500 and 600 °C, and above 600 °C were attributed to easily oxidizable α-carbon species, moderately oxidizable β-carbon species, and inert carbon species.^33^ Over 30Fe/10TiZr catalyst, the O_2_-TPO peak is broadened between 500 and 700 °C. It indicates the presence of moderately oxidizable and inert carbon species over the spent 30Fe/10TiZr catalyst upon the addition of 5 wt % Sr or Ni over 30Fe/10TiZr, and the peak widths become narrower (500–600 °C). It indicates that the population of inert carbon species is decreased over strontium or nickel-promoted 30Fe/10TiZr catalyst rather than the unpromoted catalyst; however, upon the addition of 5 wt % Co over 30Fe/10TiZr, the oxidation peak profile of the catalyst extends to higher and lower temperatures. Raman spectra of spent catalysts show the presence of defective or poorly crystalline parts of graphite (diamond carbon; D) and ordered or crystalline parts of graphite (Graphitic carbon; G) at 1340 and 1570 cm^–1^.^34,35^ The intensity index ratio of ions D and G (I_D_/I_G_) is often determined. The peak fitting of I_D_ and I_G_ is shown in Supporting Information under Figure S1. The low ratio indicates highly crystalline graphite, whereas the high ratio of I_D_/I_G_ signifies poorly crystalline graphite.^35^ The I_D_/I_G_ ratio over different catalysts is found in the following order: 30Fe5Co/10TiZr (I_D_/I_G_ = 0.93) > 30Fe5Ni/10TiZr (I_D_/I_G_ = 0.67) > 30Fe/10TiZr (I_D_/I_G_ = 0.53) > 30Fe5Sr/10TiZr (I_D_/I_G_ = 0.48). The lowest I_D_/I_G_ ratio (0.48) over the Sr promoted spent 30Fe/10TiZr catalyst indicates the presence of highly crystalline graphite over the spent catalyst surface. The O_2_-TPO analysis shows the presence of a smaller amount of oxidizable carbon species over the 30Fe5Sr/10TiZr catalyst, but the Raman analysis verifies the highest degree of crystalline graphite over the catalyst. Over the spent 30Fe5Co/10TiZr catalyst, the O_2_-TPO analysis shows the presence of the highest amount of oxidizable carbon, whereas Raman analysis revealed the presence of nearly identical amounts of graphitic and diamond carbon.

### 3.4 Discussion

By H_2_-TPR observation, it is clear that titania-modified zirconia is nonreducible, and it becomes good support for carrying active sites and promoters against high temperatures for the CH_4_ decomposition reaction. As the 30Fe/10TiZr catalyst is promoted with Sr, Co, and Ni, the Raman peaks for oxide vacancy (due to the coexistence of Fe^2+^ and Fe^3+^) are suppressed. It indicates that the oxide vacancy over 30Fe/10TiZr may be the preferred site for promoter occupancy. The deposition of promoter oxide into the pores decreases the surface area and pore volume of the promoted catalyst samples. Here, before the CH_4_ decomposition reaction, the catalyst is reduced under the H_2_ stream. The target of this pretreatment is to prepare the active sites (Fe and Ni) for the CH_4_ decomposition reaction. Here, it is observed that over the 30Fe5Sr/10TiZr catalyst, the catalytic activity is progressed after 60 min, while in the other catalyst systems (30Fe/10TiZr, 30Fe5Co/10TiZr, and 30Fe5Ni/10TiZr), the catalytic activity is progressed from the beginning of the reaction. It is reported that iron oxide, nickel oxide, or cobalt oxide can be reduced to Fe, Ni, or Co, respectively, by CH_4_, which acts as a reducing gas.^36−38^ During the reaction, the CH_4_ stream generates new catalyst active sites, which initiate fresh reactions at these sites, ultimately leading to an increase in CH_4_ conversion and H_2_ yield over time on the stream. Fe supported over titania-modified zirconia (30Fe/10TiZr) has bimodal pore distribution, monoclinic ZrO_2_ phase, rhombohedral Fe_2_O_3_ phases, anatase TiO_2_, rutile RiO_2_, and mixed oxide rhombohedral FeTiO_3_ phases. Due to the coexistence of Fe^2+^ (FeTiO_3_) and Fe^3+^ (Fe_2_O_3_), the oxygen vacancies are mounted over the catalyst surface. Catalytic pretreatment under hydrogen cultivates the catalytically active sites of “Fe” (after the reduction of iron oxide). Overall, the 30Fe/10TiZr catalyst shows an initial 65% CH_4_ conversion, 61% hydrogen yield, and 0.94 *Y*_*H*_2__/*C*_*CH*_4__ ratio. During the CH_4_ decomposition reaction, new catalytic active sites are also generated by the reduction of Fe_2_O_3_ by CH_4_. At the end of 3 h, 82% CH_4_ conversion and 77% H_2_ yield are achieved over the 30Fe/10TiZr catalyst. Upon promotional addition of 5 wt % strontium over promoted 30Fe/10TiZr, the crystallinity of the catalyst has grown, and an orthorhombic strontium zirconium oxide phase is also formed. 30Fe5Sr/10TiZr catalyst has multimodal pore size distribution. In the literature, the presence of Sr was found beneficial in attaining the active sites of “Fe” for the CH_4_ decomposition reaction.^7,11^ But here, the 30Fe5Sr/10TiZr catalyst has the same reducibility pattern as 30Fe/10TiZr. It indicates that the distribution of active sites over the 30Fe5Sr/10TiZr catalyst is about similar. Overall, the presence of strontium is not effective over the current catalyst system. Again, the presence of an inactive metal oxide over the catalyst surface may also shade the catalytic active sites. Overall, minimum activity (54% CH_4_ conversion, 50% H_2_ yield) is noticed over 30Fe5Sr/10TiZr. However, it has the highest *Y*_*H*_2__/*C*_*CH*_4__ ratio (0.94). A high *Y*_*H*_2__/*C*_*CH*_4__ ratio indicates a higher accumulation of CH_4_ or dissociated CH_4_ (CH_*x*_ species; *x* = 1–3) over the catalyst surface.^33,39^ The Raman profile shows the deposit of a highly crystalline graphite layer over the spent-30Fe5Sr/10TiZr catalyst. The highly crystalline graphite may further shade the active sites further. In the first hour of reaction, the activity is decreased continuously. After 1 h of reaction, the activity is progressed. This indicates that new active sites appeared after the reduction of iron oxide into Fe. At the end of 3 h, CH_4_ conversion, H_2_ yield, and *Y*_*H*_2__/*C*_*CH*_4__ are progressed to 56%, 58%, and 1.02, respectively. The crystallinity profile and pore size distribution profile of the cobalt-promoted 30Fe/10TiZr catalyst are very similar to those of the Sr promoted 30Fe/10TiZr catalyst. But when we compare the crystallinity of Ni-promoted 30Fe/10TiZr, it is quite different. The crystallinity of the 30Fe5Ni/10TiZr catalyst drops, and the unit cell is compressed. The reduction profiles of 30Fe5Co/10TiZr and 30Fe5Ni/10TiZr catalysts are more enriched than that of the 30Fe/10TiZr and 30Fe5Sr/10TiZr catalysts. It has reducible cobalt oxides and reducible nickel oxide phases (along with reducible iron oxide phases), which can be reduced to the respective metals. Comparing Ni and Co, metallic Ni has a stronger CH_4_ interaction energy than metallic Co.^40,41^ Overall, 30Fe5Co/10TiZr catalyst showed the lowest activity (50% CH_4_ conversion, 46% H_2_ yield, and 0.93 *Y*_*H*_2__/*C*_*CH*_4__) initially. As time progressed, the new active sites (Fe, Co) were continually formed, which geared up the catalytic activity. After 3 h, the catalytic activity of the 30Fe5Co/10TiZr catalyst exceeded that of 30Fe/10TiZr catalyst. The X-ray diffraction pattern of the Ni-promoted 30Fe/10TiZr catalyst shows a relatively lower crystallinity than the unpromoted catalyst. The lower crystallinity and enriched reducible profile indicate the better dispersion reducible species, which turn into highly dispersed catalytically active sites upon reduction. During 30–180 min, the CH_4_ conversion and H_2_ yield remain highest over Ni promoted 30Fe/10TiZr than all catalysts. At the end of 180 min, 87% CH_4_ conversion, 82% H_2_ yield, and 0.94 *Y*_*H*_2__/*C*_*CH*_4__ are achieved over the 30Fe5Ni/10TiZr catalyst.

## 4 Conclusion

Titania-modified zirconia support is nonreducible, and it can hold the catalytic active iron oxide sites and promoter metal oxide for the CH_4_ decomposition reaction. The catalyst is treated with H_2_ to prepare active sites, and metal oxide is reduced into metal by CH_4_ during the reaction. So, catalytic activity over 30Fe/10TiZr and 30Fe5M/10TiZr (M = Sr, Co, Ni) progressed from 60 min to 3 h time on stream. During 3 h TOS, 30Fe/10TiZr catalyst acquired 82% CH_4_ conversion and 77% H_2_ yield. The addition of strontium over 30Fe/10TiZr induces higher crystallinity, the highest accumulation of dissociated CH_4_ species, and the formation of crystalline graphite during the reaction, which result in inferior catalytic activity.

On the other hand, the addition of Ni or Co over 30Fe/10TiZr led to a higher density of reducible species (such as iron oxide, cobalt oxide, and nickel oxide) and active sites (formed upon the reduction of reducible species). Although 30Fe5Co/10TiZr exhibited low initial catalytic activity, it increased to over 80% within 3 h due to effective catalysis produced by the in situ Fe and Co sites generated by reduction under CH_4_. The lower crystallinity and enriched reducible profile of the 30Fe5Ni/10TiZr catalyst indicate higher dispersion of reducible species compared to the active sites formed after reduction. The activity over 30Fe5Ni/10TiZr consistently remained optimal compared to other catalysts during the time on stream of 30–180 min. At the end of 3 h, 30Fe5Ni/10TiZr achieved 87% CH_4_ conversion and 82% H_2_ yield from CH_4_ decomposition.

Overall, this research gives scientific insight into the use of dual metal oxide, titania–zirconia, for supporting iron-based catalyst, which further stabilizes the promotors (like Sr, Co, and Ni) at oxygen vacancy sites (created by Fe^2+^/Fe^3+^) for CH_4_ decomposition reaction. Further, the enriched reduction profile, in situ surging of active sites by CH_4_ during the reaction, and higher dispersion of active sites are essential parameters that need to be considered for optimum catalytic performance.

## Supporting Information Available

Peak fitting of Raman spectra of spent 30Fe/10TiZr, spent 30Fe5Sr/10TiZr, spent 30Fe5Co/10TiZr, and spent 30Fe5Ni/10TiZr (PDF)
