# Supplementary material for: Optimizing Hydrogen Production: Influence of Promoters in Methane Decomposition on Titania-Modified-Zirconia Supported Fe Catalyst
Source: ACS Omega. 2024 Apr 25;9(18):20322–30. doi: 10.1021/acsomega.4c00729 (PMC11080036; doi:10.1021/acsomega.4c00729)
Supplement: Supplementary file 1 — ao4c00729_si_001.pdf [file ao4c00729_si_001.pdf]

Supporting information for

Optimizing Hydrogen Production: Influence of Promoters in Methane Decomposition on Titania-Modified-Zirconia Supported Fe Catalyst

Ahmed Sadeq Al-Fatesh [1*], Dharmesh M. Vadodariya[2], Mohammed O. Bayazed[1], Ahmed I. Osman[3*], Ahmed Aidid Ibrahim [1], Anis Hamza Fakeeha [1], Yousef M. Alanazi[1], Ahmed E. Abasaeed[1], Rawesh Kumar[2*]

[1]Chemical Engineering Department, College of Engineering, King Saud University, P.O. Box 800, Riyadh 11421, Saudi Arabia
[2]Department of Chemistry, Indus University, Ahmedabad, Gujarat,382115, India
[3] School of Chemistry and Chemical Engineering, Queen's University Belfast, Belfast BT9 5AG, Northern Ireland, UK

*Correspondence: aalfatesh@ksu.edu.sa; osmanahmed01@qub.ac.uk ;kr.rawesh@gmail.com

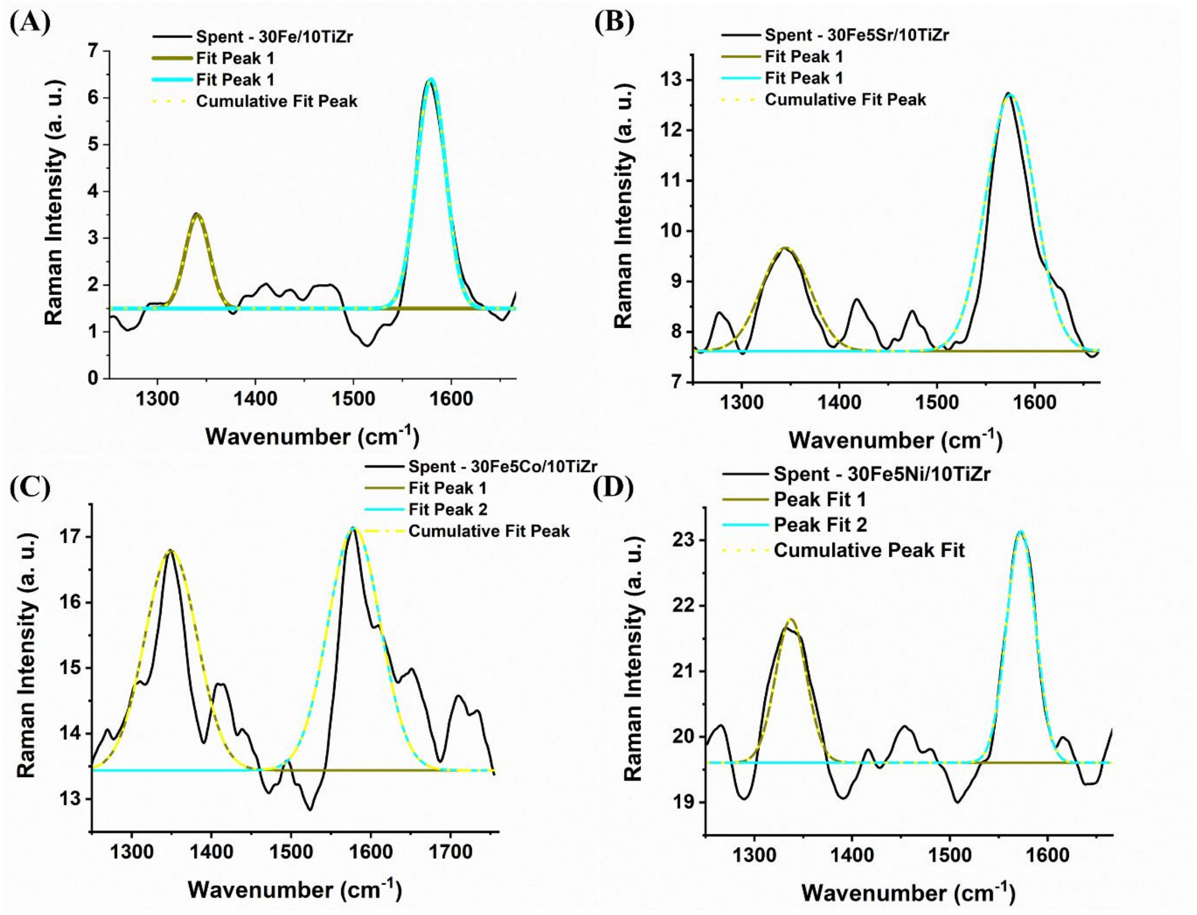

Figure S1. Peak fitting of Raman spectra of (A) spent 30Fe/10TiZr (B) spent 30Fe5Sr/10TiZr (C) spent 30Fe5Co/10TiZr (D) spent 30Fe5Ni/10TiZr
